# RACIAL AND ETHNIC DIFFERENCES IN AGEISM AND HEALTH: EVIDENCE FROM THE EXPERIENCES OF AGING IN SOCIETY PROJECT

**DOI:** 10.1093/geroni/igae098.0091

**Published:** 2024-12-31

**Authors:** Julie Ober Allen

**Affiliations:** University of Wisconsin, Madison, Wisconsin, United States

## Abstract

Age-based stereotypes, prejudices, and discrimination (ageism) are implicated in a variety of poor health outcomes, yet it is unclear if all segments of the older adult population are affected similarly. This study examined racial and ethnic differences in ageism and health using data from the Experiences of Aging in Society project (2021-2023) with a sample equally divided between NonHispanic Black, White, and Asian American and Hispanic US adults age 50+ (N=236; Mage 65; 72% female). We identified variations in amount and types of routine ageism (Everyday Ageism Scale and subscales) and associations with health using ANCOVA and z-tests of differences in race/ethnicity-stratified regression parameters. While all racial/ethnic groups reported comparable amounts of routine ageism overall, differences were detected in types: exposure to ageist messages, White=most, Black=least; age discrimination, Black=most, White=least; and internalized ageism, Hispanic=most, Black=least. In the full sample, ageism was associated with more chronic physical health conditions and greater odds of a mental health disorder (p-values<.02); of the three types, age discrimination was most closely associated with physical health outcomes and internalized ageism with mental. Relationships differed by race/ethnicity: ageism was only associated with physical health among Black (association driven by age discrimination) and White adults (driven by internalized ageism) and with mental health among White adults (driven by internalized ageism) (p-values<.05). Exposure to routine ageism may be universal; yet, healthy aging initiatives should attend to nuances at the intersection of age, race, and ethnicity that contribute to divergent experiences of ageism and consequences for mental and physical health.

